# Facile Fabrication of Large-Area CuO Flakes for Sodium-Ion Energy Storage Applications

**DOI:** 10.3390/molecules29112528

**Published:** 2024-05-28

**Authors:** Xiaolei Sun, Feng Luo

**Affiliations:** School of Materials Science and Engineering, Tianjin Key Laboratory for Rare Earth Materials and Applications, Center for Rare Earth and Inorganic Functional Materials, Nankai University, Tianjin 300350, China

**Keywords:** lithium-ion batteries, sodium-ion batteries, transition metal oxides, anode materials, two-dimensional structures, excellent electrochemical properties

## Abstract

CuO is recognized as a promising anode material for sodium-ion batteries because of its impressive theoretical capacity of 674 mAh g^−1^, derived from its multiple electron transfer capabilities. However, its practical application is hindered by slow reaction kinetics and rapid capacity loss caused by side reactions during discharge/charge cycles. In this work, we introduce an innovative approach to fabricating large-area CuO and CuO@Al_2_O_3_ flakes through a combination of magnetron sputtering, thermal oxidation, and atomic layer deposition techniques. The resultant 2D CuO flakes demonstrate excellent electrochemical properties with a high initial reversible specific capacity of 487 mAh g^−1^ and good cycling stability, which are attributable to their unique architectures and superior structural durability. Furthermore, when these CuO flakes are coated with an ultrathin Al_2_O_3_ layer, the integration of the 2D structures with outer nanocoating leads to significantly enhanced electrochemical properties. Notably, even after 70 rate testing cycles, the CuO@Al_2_O_3_ materials maintain a high capacity of 525 mAh g^−1^ at a current density of 50 mA g^−1^. Remarkably, at a higher current density of 2000 mA g^−1^, these materials still achieve a capacity of 220 mAh g^−1^. Moreover, after 200 cycles at a current density of 200 mA g^−1^, a high charge capacity of 319 mAh g^−1^ is sustained. In addition, a full cell consisting of a CuO@Al_2_O_3_ anode and a NaNi_1/3_Fe_1/3_Mn_1/3_O_2_ cathode is investigated, showcasing remarkable cycling performance. Our findings underscore the potential of these innovative flake-like architectures as electrode materials in high-performance sodium-ion batteries, paving the way for advancements in energy storage technologies.

## 1. Introduction

In alignment with global frameworks for achieving sustainable development, there is an increasing dependency on renewable energy sources [1,2,3]. This trend has accelerated the advancement of cost-effective and sustainable energy storage solutions [1,4]. Among these, sodium-ion batteries (SIBs) have emerged as a promising option for large-scale energy storage due to their abundant availability (~2.74 wt.% versus 0.0017 wt.% for Li), economical production costs, and reaction mechanism akin to lithium-ion batteries (LIBs) [5,6]. However, the graphite anodes commonly employed in LIBs are inefficient in SIBs because of their lower capacity and unsuitable thermodynamic properties [7]. Consequently, many efforts have been made to identify high-performance anode materials for SIBs, including hard carbon, alloys, metal oxides, and sulfides [8,9,10,11]. The considerable radius of sodium ions (0.106 nm) compared with that of lithium ions (0.076 nm) presents notable challenges, slowing kinetic processes and undermining the structural integrity of host materials [12,13]. Nevertheless, morphological and structural modifications through nanoengineering and carbon adjustments of anode materials offer promising avenues for enhancing the mechanical stability and sodium storage kinetics of SIBs [14,15,16,17]. These innovative material designs are crucial for enhancing sodium storage efficiency and reaction kinetics, which are critical to the development and practical use of SIB technology [18,19].

Recent initiatives have focused on developing electrodes with higher capacities, spurring significant interest in micro/nanomaterials that offer larger surface areas and reduced ion and electron transport distances compared with those of bulk materials [20,21]. Cupric oxide (CuO), in particular, has garnered attention as a potential anode material because of its high theoretical capacity based on conversion reactions (674 mAh g^−1^), affordability, and chemical robustness [22,23,24]. Despite these advantages, the practical deployment of CuO and other metal oxides is impeded by their inherent poor conductivity and substantial volumetric expansion over repeated discharge/charge cycles, which could result in significant capacity loss and diminished performance rates [25,26]. Prior modifications to CuO micro/nanostructures have yielded only limited advancements in their electrochemical stability, except when they are integrated with a dense carbon matrix [27,28,29,30]. For example, Wen et al. fabricated hollow and hierarchical CuO micro-nano cubes wrapped with reduced graphene oxide via a wet-chemical method for use as anode materials in SIBs, achieving a reversible specific capacity of 218.9 mAh g^−1^ after 150 cycles at a current density of 300 mA g^−1^ [31]. Zhang and colleagues developed carbon nanofibers decorated with Cu/CuO nanoparticles through magnetron sputtering and achieved an initial Coulombic efficiency of 76% and a reversible specific capacity of 300 mAh g^−1^ after 400 cycles at a current density of 100 mA g^−1^ [32]. Chen’s group introduced a freestanding anode made of nitrogen-doped carbon-coated CuO arrays, which showcased superior electrochemical performance with a reversible specific capacity of 214.97 mAh g^−1^ following 100 cycles at a current density of 500 mA g^−1^, outperforming the bare CuO arrays anode (183.21 mAh g^−1^) [20]. However, attaining the requisite long-term cyclability and high-rate performance for commercial applications continues to pose a significant challenge.

In recent research endeavors, the exploration of two-dimensional (2D) structures, such as nanosheets, nanoflakes, and nanoplates, has been highlighted because of their potential to enhance electrochemical energy storage systems [33,34,35,36]. These 2D architectures offer advantages by facilitating accelerated conversion reaction kinetics, attributable to their larger surface areas and shortened pathways for electron and ion transport [37,38]. However, significant energy storage in these structures is typically achieved only under high temperatures or low current densities, often followed by a rapid decline in capacity [7]. This reduction in capacity over time is speculated to be due to substantial material expansion and contraction, potentially causing electrode fracture or disintegration, leading to rapid capacity degradation [27]. Moreover, the direct contact between the electrode and the electrolyte can lead to the continuous formation of solid electrolyte interphase (SEI) layers on fractured surfaces, further diminishing the Coulombic efficiency [7]. Thus, it is imperative to develop effective strategies that can shield electrode surfaces from electrolytes during discharge/charge cycles. Several technologies, including atomic layer deposition (ALD), magnetron sputtering, and other physical or chemical deposition methods, have been developed for electrode surface modification [20,39,40,41]. Notably, ALD has emerged as a particularly promising technique for electrode surface modification, significantly reducing direct electrode/electrolyte contact, thereby minimizing side reactions and substantially prolonging the cycle life [40]. Aluminum oxide (Al_2_O_3_) has emerged as the predominant coating material in numerous studies, owing to its extensive application in both academic research and industrial settings [42,43]. Therefore, the design of a 2D flake-like CuO structure combined with an ultrathin conformal Al_2_O_3_ coating layer could be a promising strategy for achieving desirable sodium storage.

Inspired by the aforementioned postulation, herein, we successfully fabricated large-area 2D CuO and CuO@Al_2_O_3_ flakes through a combination of magnetron sputtering deposition, thermal oxidation, and Al_2_O_3_ ALD. When applied as sodium storage materials, the designed 2D CuO flakes exhibit superior electrochemical properties with a high initial reversible capacity of 487 mAh g^−1^ and good cycling stability, which are attributable to their unique architectures and superior structural durability. Furthermore, as expected, when the CuO flakes are coated with an ultrathin Al_2_O_3_ layer, the electrochemical properties are improved greatly. Specifically, after undergoing 70 rate testing cycles, the CuO@Al_2_O_3_ materials still exhibit a high specific capacity of 525 mAh g^−1^ at a current density of 50 mA g^−1^. Even at a higher current density of 2000 mA g^−1^, a notable capacity of 220 mAh g^−1^ is achieved. Following 200 cycles at a current density of 200 mA g^−1^, they can maintain a high specific charge capacity of 319 mAh g^−1^. Moreover, the full cell comprising a CuO@Al_2_O_3_ anode and a NaNi_1/3_Fe_1/3_Mn_1/3_O_2_ cathode, exhibiting impressive cycle stability, underscores the significant application potential of CuO flakes as SIB anodes. The present work is expected to offer a promising strategy for the rational design and production of other advanced metal oxide-based materials for high-performance SIBs.

## 2. Results and Discussion

**Figure 1:**
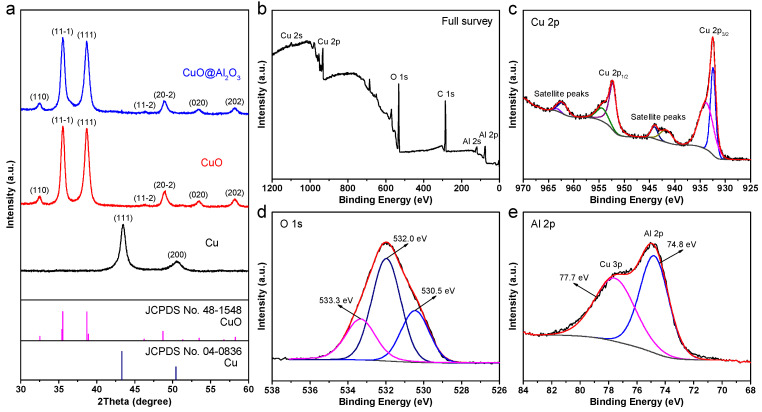
(**a**) XRD patterns of the as-prepared Cu, CuO, and CuO@Al_2_O_3_ products. (**b**) XPS survey spectrum and high-resolution spectra of (**c**) Al 2p, (**d**) Cu 2p, and (**e**) O 1s of the CuO@Al_2_O_3_ composite.

The detailed fabrication processes for the large-area CuO and CuO@Al_2_O_3_ flakes are described in the Experimental Section. Briefly, the process began with the production of Cu flakes via magnetron sputtering onto a substrate pre-coated with a sacrificial photoresist layer, which was later eliminated through acetone etching. The as-obtained Cu flakes were then annealed at 600 °C for 3 h in an air atmosphere, triggering an oxidation process that naturally formed CuO flakes (Cu + O_2_ → CuO). Subsequently, the CuO@Al_2_O_3_ flakes could be obtained by carefully depositing a thin and consistent Al_2_O_3_ layer onto the CuO flakes using the ALD technique. Figure 1a illustrates the X-ray diffraction (XRD) patterns of the as-prepared Cu, CuO, and CuO@Al_2_O_3_ samples. Clearly, the pristine Cu specimen displays two distinct diffraction peaks in the black curve at 43.5° and 50.6°, corresponding to the (111) and (200) crystal planes of the cubic Cu phase (JCPDS No. 04-0836), respectively. After heat treatment, the diffraction peaks in the red curve observed at 32.5°, 35.5°, 38.7°,46.3°, 48.8°, 53.4°, and 58.2° can be assigned to the (110), (11-1), (111), (11-2), (20-2), (020), and (202) crystal planes of the monoclinic CuO phase (JCPDS No. 48-1548), respectively. The absence of sharp peaks corresponding to other impurities suggests the successful conversion of Cu flakes into pure-phase CuO flakes through the thermal oxidation process. Furthermore, the XRD pattern of the CuO@Al_2_O_3_ composite in the blue curve aligns with that of the bare CuO material, suggesting that the Al_2_O_3_ coating process does not modify the CuO crystal structure. The absence of distinct diffraction peaks for the Al_2_O_3_ phase is likely due to its minimal mass contribution and the existence of an amorphous structure, which precludes the formation of discrete diffraction patterns. In order to evaluate the complete coating of CuO by the Al_2_O_3_ nanolayer, the surface valence states and elemental composition of the CuO@Al_2_O_3_ composite were examined using X-ray photoelectron spectroscopy (XPS). The XPS survey spectrum (Figure 1b) indicates that the surface of the CuO@Al_2_O_3_ composite contains Cu, Al, and O elements. Thus, the detection of signals from Cu, along with the average depth (less than 10 nm) of the XPS technique, confirms the ultrathin nature of the Al_2_O_3_ coating layer, which is consistent with earlier XRD findings (Figure 1a). The high-resolution XPS spectrum of Al 2p, illustrated in Figure 1c, features a pronounced peak at 74.8 eV, which signifies the presence of Al 2p originating from the Al_2_O_3_ coating [44]. Further analysis of the high-resolution XPS spectrum of Cu 2p, presented in Figure 1d, reveals two distinct peaks at binding energies of 952.3 and 932.5 eV, aligning with the Cu 2p_1/2_ and Cu 2p_3/2_ peaks associated with oxidized copper(II) species [45]. Additionally, satellite peaks observed in the ranges of 965.1 to 959.5 eV and 945.8 to 939.6 eV verify the existence of the 3d^9^ shell of the Cu^2+^ state [31]. The spin-orbit coupling energy difference of approximately 19.8 eV between the Cu 2p_1/2_ and Cu 2p_3/2_ peaks aligns well with the characteristics of the Cu^2+^ oxidation state [15,31]. In the high-resolution XPS spectrum of O 1s shown in Figure 1e, the peak at 530.5 eV is attributable to lattice oxygen, whereas the peaks at 532.0 and 533.3 eV are linked to oxygen vacancies and surface-adsorbed oxygen, possibly from the adsorption of oxygen-containing molecules in the air [26,31]. Therefore, the multi-step preparation process yields a CuO@Al_2_O_3_ composite consisting of both CuO and Al_2_O_3_ components.

**Figure 2:**
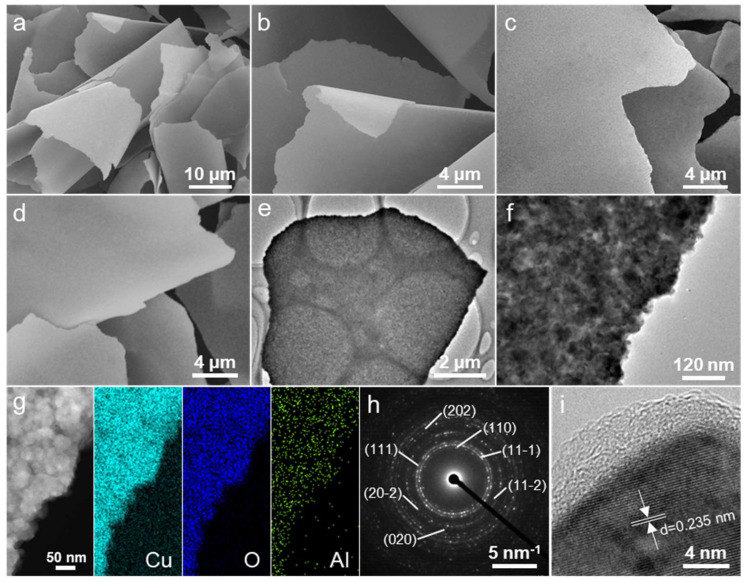
SEM images of the as-prepared (**a**,**b**) Cu, (**c**) CuO, and (**d**) CuO@Al_2_O_3_ flakes. (**e**,**f**) TEM images, (**g**) DF-STEM image and corresponding elemental mapping images of Cu, Al, and O, (**h**) SAED pattern, and (**i**) high-resolution TEM image of a single CuO@Al_2_O_3_ flake.

The scanning electron microscopy (SEM) images presented in Figure 2a–d offer an in-depth examination of the morphological characteristics of the prepared Cu, CuO, and CuO@Al_2_O_3_ flakes, highlighting the distinct transformations these materials underwent through various treatment processes. Figure 2a,b depict the initial state of the Cu flakes, characterized by their flake-like morphology with a significant microscale lateral dimension contrasted against their thinness, approximately 150 nm in thickness. This stark difference in the dimensions endows these materials with a notably high aspect ratio, potentially influencing their mechanical properties. Upon subjecting these Cu flakes to calcination at 600 °C for 3 h in an air atmosphere, a remarkable transformation occurs, as observed in Figure 2c. The resultant CuO flakes retain their original flake-like shape; however, a significant change in surface texture is evident, transitioning from a smooth to a more textured surface. Furthermore, the thickness of the CuO flakes is found to increase to about 250 nm as a result of the oxidation process. A comparison of the SEM images of the CuO flakes (Figure 2c) with those of the CuO@Al_2_O_3_ flakes (Figure 2d) reveals a consistent surface roughness despite the application of the ALD process. This observation suggests that the ALD coating is exceedingly thin, potentially because it is electron-transparent under SEM conditions. This consistency in surface texture following the ALD treatment, which was conducted at a relatively mild temperature of 200 °C, underscores the capability of the process to coat without significantly altering the fundamental structural and morphological characteristics of the base material, preserving the integrity of the flake-like morphology while potentially enhancing its functional properties. As expected, the as-prepared CuO and CuO@Al_2_O_3_ flakes have notably high Brunauer–Emmett–Teller (BET) specific surface area values with abundant accessible reactive sites, as shown in Table 1.

Transmission electron microscopy (TEM) observation was further conducted after sonicating the CuO@Al_2_O_3_ material for 5 min. TEM images at various magnifications (Figure 2e,f) confirm that after sonication, the CuO@Al_2_O_3_ composite maintains its flake-shaped morphology on a Cu grid, featuring rough surfaces attributable to the presence of numerous tiny particles. This kind of ultra-thin material, distinguished by its large flake-like structure, may hold promising potential for substantially bolstering sodium storage capabilities by minimizing ion diffusion distances, thus facilitating enhanced ion intercalation kinetics and overall energy storage efficiency. Furthermore, the corresponding dark-field scanning TEM (DF-STEM) image and the energy-dispersive X-ray (EDX) elemental mapping images (Figure 2g) reveal the presence of copper (Cu), aluminum (Al), and oxygen (O) elements with homogeneous distributions in the CuO@Al_2_O_3_ flake. Additionally, the semi-quantitative EDX analysis reveals the presence of approximately 0.21 wt.% Al_2_O_3_ in the CuO@Al_2_O_3_ composite. Furthermore, the selected area electron diffraction (SAED) pattern (Figure 2h) reveals a series of diffraction rings, indicative of the polycrystalline nature of the composite. These rings can be ascribed to the monoclinic structure of CuO, specifically corresponding to the diffractions of the (110), (11-1), (111), (11-2), (20-2), (020), and (202) planes. These findings align well with the prominent peaks observed in the XRD pattern. The absence of information on the Al_2_O_3_ phase indicates that the amorphous Al_2_O_3_ surface layer is too thin for detection. Based on the high-resolution TEM image displayed in Figure 2i, the lattice spacings of 0.235 nm can be attributed to the (111) planes of the CuO phase. Additionally, it is evident that the entire surface of the nanoflake is uniformly covered with a continuous amorphous Al_2_O_3_ overlayer with a thickness of approximately 4 nm, which aligns with the expected growth rate achieved through the ALD technique.

**Figure 3:**
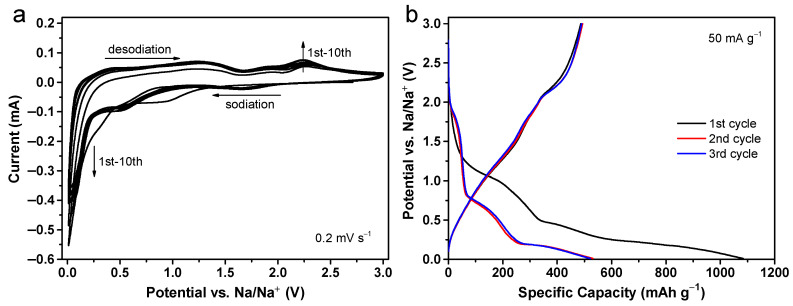
(**a**) CV curves for the top ten cycles of the CuO@Al_2_O_3_ flakes electrode ranging from 0.01 to 3 V versus Na/Na^+^ at a scan rate of 0.2 mV s^−1^. (**b**) Galvanostatic discharge/charge profiles for the first three cycles of the CuO@Al_2_O_3_ flakes electrode at a current density of 50 mA g^−1^.

To evaluate the sodium storage properties, Swagelok-type cells were constructed for both CuO and CuO@Al_2_O_3_ flakes, and various electrochemical tests were conducted at ambient temperature. The cyclic voltammetry (CV) profiles of the top ten cycles for the CuO@Al_2_O_3_ flakes within a voltage range of 0.01 to 3 V versus Na/Na^+^ at a scan rate of 0.2 mV s^−1^ are depicted in Figure 3a. In the initial cathodic scan process (sodiation), two distinct cathodic peaks at 0.95 and 0.29 V are detected, which are associated with several reversible electrochemical transformations [46]. According to existing studies, these reactions may include the formation of an intermediate copper oxide state Cu_1−*x*_^II^Cu*_x_*^I^O_1−*x*/2_, the generation of a Cu_2_O phase, and subsequent reduction of the Cu_2_O phase to Cu and Na_2_O [22,47]. Additionally, there is some irreversible decomposition of the organic electrolyte, leading to the formation of an SEI layer within this voltage range [15]. Starting from the second cycle, the reduction peaks shift to higher potentials, approximately ∼1.74 and ∼0.48 V. In the anodic scan (desodiation) process, several oxidation peaks at 0.51, 1.22, 1.94, and 2.26 V are observed. The minor peaks at 0.51 and 1.22 V are attributable to the oxidation of a limited amount of residual Cu_2_O back to CuO, while the peak at 1.94 V is linked to the reformation of Cu_2_O from Cu [22]. The fourth peak at 2.26 V is related to the oxidation of Cu_2_O into the CuO phase [12,22]. In subsequent cycles, the observed significant changes in the cathodic peaks on the CV curves indicate a sodium-induced activation process. In other words, the peak intensity of the current increases progressively in each cycle, indicating an increasing amount of material becomes active and interacts with sodium in successive scans. This type of activation is often seen in oxide electrodes and typically results in increased capacity through subsequent electrochemical cycles because of its ability to enhance reaction kinetics [40]. Such activation can be triggered by various mechanisms, including changes in the structure of the electrode, the presence of crystalline defects, and the formation of a surface SEI layer [40].

The galvanostatic discharge/charge voltage profiles over the initial three cycles for the CuO@Al_2_O_3_ flakes electrode, conducted at a current density of 50 mA g^−1^ within a voltage range of 0.01 to 3 V, are illustrated in Figure 3b. These profiles exhibit potential plateaus that correlate with the CV observations for both the initial and subsequent cycles. Moreover, the initial discharge and charge cycles yield specific capacities of 1084 and 487 mAh g^−1^, respectively. The obvious loss in capacity can primarily be ascribed to irreversible reactions such as the incorporation of sodium ions into the CuO lattice, the formation of SEI components, and the decomposition of electrolyte molecules, phenomena commonly associated with conversion-type anode materials [7,12]. Moreover, it is essential to consider that the irreversible capacity loss could pose challenges to practical applications. Therefore, additional exploration is needed to assess the effectiveness of pre-sodiation techniques, which alter the anodic surface to enhance the initial Coulombic efficiency [48]. Notably, there is a rapid increase in Coulombic efficiency to 92.8% and 93.9% during the second and third cycles, respectively, with the discharge/charge profiles showing significant overlap, thereby reinforcing the reversibility of the sodium storage mechanisms involved.

**Figure 4:**
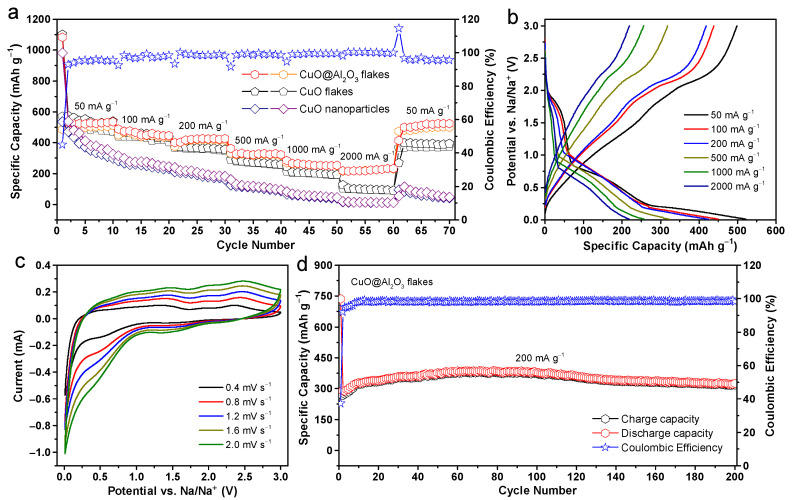
(**a**) Rate capabilities at various current densities from 50 to 2000 mA g^−1^ of the CuO nanoparticles, CuO, and CuO@Al_2_O_3_ flakes electrodes. (**b**) Discharge/charge profiles of the CuO@Al_2_O_3_ flakes electrode at different current densities. (**c**) CV profiles of the CuO@Al_2_O_3_ flakes electrode at different scan rates ranging from 0.4 to 2 mV s^−1^. (**d**) Cycling performance and the corresponding Coulombic efficiency plots of the CuO@Al_2_O_3_ flakes electrode at a current density of 200 mA g^−1^.

The rate capabilities of the CuO and the CuO@Al_2_O_3_ flakes, along with the commercial CuO nanoparticles, were investigated for ten cycles each at various current densities ranging from 50 to 2000 mA g^−1^. As presented in Figure 4a, the CuO@Al_2_O_3_ flakes demonstrate superior specific capacities in comparison to those of the CuO nanoparticles, similar to the CuO flakes without the Al_2_O_3_ coating, particularly at high discharge and charge current densities. At a lower current density of 50 mA g^−1^, the specific charge capacity recorded for the CuO@Al_2_O_3_ flakes is 499 mAh g^−1^, aligning with the performance observed for CuO flakes under identical conditions. The sodium storage performance of the CuO@Al_2_O_3_ flakes is notably enhanced as the current density for discharge and charge increases to 100, 200, 500, and 1000 mA g^−1^. At these current densities, the CuO@Al_2_O_3_ flakes exhibit specific capacities of 439, 419, 319, and 257 mAh g^−1^, respectively. In contrast, under the respective current densities, the pure CuO flakes demonstrate significantly lower specific capacities of 436, 358, 270, and 202 mAh g^−1^, respectively. Even at an extremely high current density of 2000 mA g^−1^, the CuO@Al_2_O_3_ flakes maintain a high specific capacity of 220 mAh g^−1^ over 55 cycles, significantly outperforming the CuO flakes and CuO nanoparticles, which only exhibit capacities of 96 and 13 mAh g^−1^, respectively. Remarkably, upon reducing the current density back to 50 mA g^−1^ and subjecting it to an additional 10 cycles of continuous cycling, the CuO@Al_2_O_3_ flakes electrode exhibits a remarkable discharge capacity of 525 mAh g^−1^, corresponding to about 98.7% of the discharge capacity in the second cycle and over 95.8% Coulombic efficiency after 70 cycles. Meanwhile, the CuO flakes electrode exhibits gradual capacity fading, retaining only about 375 mAh g^−1^ under the same testing conditions. Overall, the sodium storage capability of the CuO flakes is marginally inferior to that of the CuO@Al_2_O_3_ flakes but significantly surpasses that of the CuO nanoparticles. This improvement suggests that the ALD Al_2_O_3_ coating enhances the rate capability and cycling performance of the CuO@Al_2_O_3_ flakes, enabling them to sustain high-current operation more effectively.

Figure 4b displays the corresponding voltage behaviors under different high current conditions for the CuO@Al_2_O_3_ flakes electrode, showing a lower voltage during discharge and a higher voltage during charging as the current density increases. This behavior is associated with the kinetic characteristics of the battery materials, consistent with findings from earlier research [34,49]. The electrochemical reaction dynamics of the CuO@Al_2_O_3_ flakes electrode were further analyzed through CV curves at various scan rates. Figure 4c shows that within a potential range of 0.01 to 3 V, the electrode consistently exhibits reliable redox behavior over scan rates from 0.4 to 2 mV s^−1^, demonstrating its excellent rate capability. An increase in polarization with increasing scan rate causes the oxidation and reduction peaks to shift towards higher and lower potential values, respectively. However, the overall shape of the CV curve remains relatively unchanged, suggesting the high stability and fast electrochemical response of the CuO@Al_2_O_3_ flakes electrode. The apparent Na^+^ diffusion coefficient at room temperature can be calculated by using the Randles–Ševčík equation: *i*_p_ = 2.69 × 10^5^ *n*^3/2^*CAD*^1/2^*v*^1/2^, where the *i*_p_ represents the peak current, *n* is the number of electrons per reaction species, *C* is the concentration of Na^+^ in the lattice, *A* is the area of electrode, *D* is the apparent Na^+^ diffusion coefficient, and *v* stands for the scan rate [50]. Upon fitting to this equation, the CuO@Al_2_O_3_ anode manifests notably high diffusion coefficients, with values of approximately 2.6 × 10^−14^ cm^2^ s^−1^ for sodiation and 1.5 × 10^−14^ cm^2^ s^−1^ for desodiation, respectively, implying good electrochemical kinetic properties.

The CuO@Al_2_O_3_ flakes electrode not only demonstrates an excellent rate capability but also shows exceptional cycling performance. When subjected to continuous cycling at a current density of 200 mA g^−1^ for 200 cycles, the anode composed of CuO@Al_2_O_3_ flakes demonstrates superior cyclability, as illustrated in Figure 4d. During the first 100 cycles, the specific charge capacity of the CuO@Al_2_O_3_ flakes steadily rises from 275 to 373 mAh g^−1^. This enhancement could be attributed to the activation of the electrode materials, which aligns with the findings from CV studies and corroborates with existing reports in the scientific literature [37,51]. However, further studies are needed to understand the underlying mechanisms thoroughly. The anode consistently maintains a high charge capacity beyond 100 cycles, achieving 319 mAh g^−1^ at the 200th cycle. Obviously, except for the first few cycles, the Coulombic efficiency consistently exceeds 99.5% throughout the subsequent discharge/charge processes. Additionally, Table 2 provides a comprehensive comparison of the sodium storage performance among different CuO-based anodes with varying structural designs, as documented in the recent literature. The achieved electrochemical performance notably exceeds that of many advanced CuO materials previously reported, such as CuO nanoparticles [15], hollow nanocubes [52], and porous microspheres [53,54], highlighting the superior sodium-storage capabilities of the CuO@Al_2_O_3_ flakes developed in this study.

**Figure 5:**
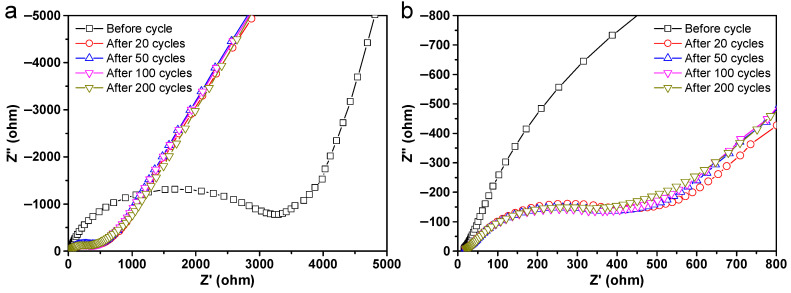
(**a**) Nyquist plots and (**b**) enlarged Nyquist plots of the CuO@Al_2_O_3_ flakes electrode before cycling and after 20, 50, 100, and 200 cycles.

To investigate the electrochemical reaction kinetics of the CuO@Al_2_O_3_ flakes electrode, cell impedances before and after a specified number of cycles are compared in Figure 5. All impedance measurements were made at the fully charged state. Figure 5a illustrates that the Nyquist plots depict the highest charge transfer resistance of the pristine electrode, signifying its state before undergoing discharge/charge processes. The total SEI and charge transfer resistances slightly decrease during the initial 100 cycles, although they remain significantly lower than the initial value (Figure 5b). The gradual decrease in electrochemical reaction resistances likely results from the activation of surface and lattice expansion mechanisms, facilitating sodium ion transport and indicating a gradual increase in specific capacities during this cycling period [22,46]. At the 200th cycle, the resistance increase could be attributable to electrode polarization, indicating physicochemical changes at the electrode surface that gradually degrade battery performance [56]. Importantly, the CuO@Al_2_O_3_ flakes electrode exhibits minimal resistance variation with increasing cycle numbers, indicating the robustness of the SEI layer formed during the initial battery cycle. This phenomenon enhances electron kinetics within the electrode, consistent with observations from electrochemical experiments.

**Figure 6:**
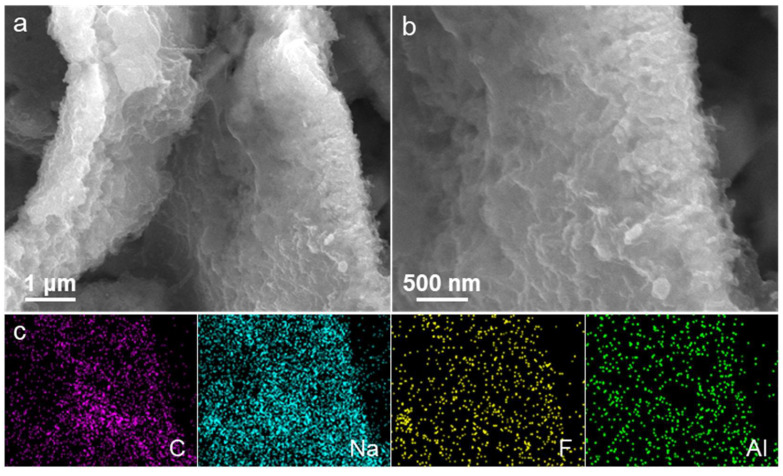
SEM images at (**a**) high and (**b**) low magnification, along with (**c**) the corresponding elemental mapping images for C, Na, F, and Al of the CuO@Al_2_O_3_ flakes electrode after undergoing 200 discharge/charge cycles at a current density of 200 mA g^−1^.

To investigate the structural changes in the CuO@Al_2_O_3_ flakes after extensive cycling tests, the electrode containing these flakes was dismantled following 200 discharge/charge cycles at a current density of 200 mA g^−1^ and subsequently cleaned with dimethyl carbonate. The SEM images taken at different magnifications (Figure 6a,b) reveal that the cycled CuO@Al_2_O_3_ flakes largely maintain their large surface area and characteristic flake-like morphology even after 200 cycles. Notably, the thickness of the flakes has increased compared with that of the pristine flakes, as depicted in Figure 2d, suggesting that some material accumulated or transformed during long-term cycling. The surface of the flakes seems to have acquired additional thin coatings, which are mainly composed of uniform polymeric gel-like layers, distinct from the initial surface. This is further supported by the elemental mapping presented in Figure 6c, which demonstrates a uniform distribution of elements such as carbon (C), Na (sodium), F (fluorine), and aluminum (Al) across the flake surface. This uniform dispersion suggests the development of a consistent SEI layer on the cycled CuO@Al_2_O_3_ flakes electrode, which could play a role in its cycling stability and rate performance. Consequently, the consistent formation of the SEI layer, along with the maintained structural integrity, not only underscores the potential of CuO@Al_2_O_3_ flakes as high-performance electrode materials for SIBs but also offers pivotal guidance for the further optimization of electrode design and the enhancement of battery efficiency.

**Figure 7:**
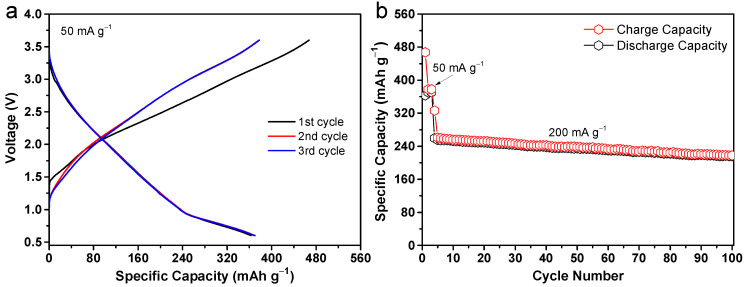
Electrochemical performance of a typical full sodium-ion battery constructed with CuO@Al_2_O_3_ anode and NaNi_1/3_Fe_1/3_Mn_1/3_O_2_ cathode. (**a**) Galvanostatic charge/discharge profiles and (**b**) cycling performance over 100 cycles.

The favorable electrochemical performance exhibited by the CuO@Al_2_O_3_ flakes in the half battery motivates us to explore their potential in full battery systems. Figure 7 illustrates the cycling performance of a full cell consisting of a CuO@Al_2_O_3_ anode and a NaNi_1/3_Fe_1/3_Mn_1/3_O_2_ cathode. The cell was initially tested at a current density of 50 mA g^−1^ for the first 3 cycles, followed by continuous cycling at a higher current density of 200 mA g^−1^ for up to 100 cycles. Figure 7a shows the typical charge/discharge curves of the CuO@Al_2_O_3_/NaNi_1/3_Fe_1/3_Mn_1/3_O_2_ full cell cycled at a current density of 50 mA g^−1^ within a potential ranging from 0.6 to 3.6 V. As evident from the results, the full cell delivers an initial discharge capacity of 363 mAh g^−1^ based on the mass of the CuO@Al_2_O_3_ anode with an average output of approximately 1.8 V. Even at the 100th cycle under a higher current density of 200 mA g^−1^, the full cell maintains a discharge capacity of 216 mAh g^−1^ without noticeable capacity degradation (Figure 7b). As anticipated, the results align with the notion that the full battery represents a more intricate system compared with its half-battery counterpart. Therefore, these findings suggest that the CuO@Al_2_O_3_ flakes might hold significant promise for sodium storage applications.

## 3. Materials and Methods

### 3.1. Materials Preparation

Synthesis of CuO flakes: Initially, a silicon substrate was uniformly coated with a positive resist (JASP-41) through spin coating for 30 s. This step was succeeded by thermal treatment at 90 °C for 3 min to ensure the solidification of the resist layer. Thereafter, the substrate was exposed to radio frequency magnetron sputtering (JCP500, Technol, Beijing, China) to facilitate the deposition of a Cu layer. The deposition ambiance was controlled with argon gas at a pressure of 1.1 Pa, and sputtering was performed with the power of the Cu target set at 80 W for 5 min. Following Cu layer deposition, a selective etching process using acetone was applied to dissolve the resist, leading to the isolation of Cu flakes. These flakes were then meticulously cleaned with isopropyl alcohol and deionized water, followed by freeze-drying at −20 °C under vacuum conditions for 72 h. The resultant Cu flakes were subsequently heated at 600 °C in the air for 3 h at a ramp rate of 10 °C min^−1^ and allowed to cool naturally to room temperature, enabling the transformation of the metallic Cu to CuO flakes.

Coating CuO flakes with Al_2_O_3_ nanolayers (CuO@Al_2_O_3_): The application of ultrathin Al_2_O_3_ layers onto CuO flakes was achieved through the use of a specialized ALD system under a base pressure of 1 Pa. Following the standard procedure, 200 mg of CuO powder was evenly spread in an aluminum alloy tray and introduced into the ALD chamber. Nitrogen gas was utilized as both the carrier and purge gas, with a constant flow rate of 50 sccm. Trimethylaluminum (TMA) and deionized water, both of which are stored at ambient temperature, were used as precursors. The ALD chamber temperature was maintained at 200 °C, and the precursor delivery lines were kept at 90 °C. The ALD cycle involved a 0.1-s TMA pulse, a 20-s interval, a 35-s N_2_ purge, a subsequent 0.1-s deionized water pulse, another 20-s interval, and a final 35-s N_2_ purge. The reaction sequences for depositing a single atomic layer of Al_2_O_3_ through an ALD process utilizing TMA and H_2_O are as follows: (1) AlOH* + Al(CH_3_)_3_ → AlO − Al(CH_3_)_2_* + CH_4_ and (2) AlCH_3_* + H_2_O → Al − OH* + CH_4_. The experimental findings indicated that the growth rate of the Al_2_O_3_ layer after 40 ALD cycles was approximately 0.1 nm per cycle, demonstrating the efficiency of the ALD technique in depositing uniform nanocoatings.

### 3.2. Materials Characterization

X-ray powder diffraction (XRD) measurements were conducted utilizing a Rigaku SmartLab diffractometer (Rigaku Corporation, Tokyo, Japan) within the 2θ range of 20 to 60 degrees. This apparatus was equipped with a filtered Cu Kα radiation source (λ = 0.15418 nm) and was operated at a tube voltage of 40 kV and a tube current of 40 mA. For X-ray photoelectron spectroscopy (XPS), an ESCALAB 250Xi spectrometer (Thermo Scientific, Waltham, MA, USA) was utilized. Photoemission was initiated employing a 300 W monochromatic Al Kα X-ray source, exhibiting a photon energy of 1486.6 eV. The energy analyzer was set to a constant pass energy of 100 eV for survey scans, with a step size of 1 eV, and 20 eV for high-resolution scans, with a step size of 0.05 eV for core level lines. The calibration of all binding energy positions was achieved using the C 1s peak located at 284.8 eV from surface adventitious carbon. Before the XPS analysis, the samples were subjected to a drying process at 110 °C in a vacuum oven for 24 h to remove any surface moisture. The specific surface areas of the as-synthesized products were determined by nitrogen adsorption/desorption measurements on an automated gas adsorption analyzer (Micromeritics ASAP 2460, Norcross, GA, USA). The examination of sample morphologies was carried out using a JSM-7800 field-emission scanning electron microscope (SEM, 15 kV, JEOL Ltd., Tokyo, Japan) and a JEM-2800 transmission electron microscope (TEM, 200 kV, JEOL Ltd., Tokyo, Japan), both of which were equipped with an energy dispersive X-ray (EDX) analyzer.

### 3.3. Electrochemical Measurements

To explore the electrochemical behaviors associated with sodium storage, the synthesized samples were utilized as electrodes within Swagelok-type cells, assembled inside a glove box filled with argon. The electrodes were composed of active materials, conductive multi-walled carbon nanotubes, and sodium alginate binder at a weight ratio of 80:10:10 and were homogenized in deionized water. This slurry was uniformly spread over copper foil using a doctor blade technique. Following the initial water evaporation, the copper foil coated with the active materials was subjected to drying at 105 °C in a vacuum environment for 12 h. The loading amount of active materials (CuO and CuO@Al_2_O_3_) for all electrodes was 1.0 ± 0.1 mg cm^−2^. Metallic sodium foil served as both the counter and reference electrode, while the glass fiber membrane functioned as the separator. For full cell tests, commercial aluminum foil coated with sodium nickel iron manganese oxide (NaNi_1/3_Fe_1/3_Mn_1/3_O_2_) was selected instead of sodium foil. In particular, CuO@Al_2_O_3_ anodes underwent pre-sodiation in half-cells for 3 cycles to mitigate the irreversible capacity loss before being re-assembled into full cells. The electrolyte solution comprised 1 M NaPF_6_ dissolved in a solvent mixture of ethylene carbonate (EC), dimethyl carbonate (DMC), and ethyl methyl carbonate (EMC) in equal volume ratios. It is worth noting that a consistent volume of 100 μL of electrolyte was injected into each cell using a microsyringe. Electrochemical analyses were conducted using an electrochemical workstation (CHI660E, CHI Instruments, Shanghai, China), which facilitated the collection of cyclic voltammetry (CV) data ranging from 0.01–3 V versus Na/Na^+^ at a scan rate of 0.1 mV s^−1^, as well as electrochemical impedance spectroscopy (EIS) spectra from 100 kHz to 0.01 Hz. Galvanostatic discharge/charge tests were performed utilizing a multichannel battery tester (CT3002A, LANHE, Wuhan, China) within voltage ranges of 0.01–3 V (for half cells) and 0.6–3.6 V (for full cells) at various current densities. All the above-mentioned electrochemical tests were conducted at room temperature. It should be noted the specific capacities were determined by considering the weights of both anodic CuO and CuO@Al_2_O_3_ materials, with the mass of Al_2_O_3_ included in the total calculation.

## 4. Conclusions

In summary, we developed a novel fabrication strategy to prepare large-area CuO and CuO@Al_2_O_3_ flake structures, utilizing a combination of magnetron sputtering deposition, thermal oxidation, and Al_2_O_3_ ALD. The thin flake design significantly reduces electron/sodium ion transport pathways, effectively mitigating the volumetric expansion issue in CuO during discharge/charge cycles, which is known to cause rapid capacity degradation. The application of Al_2_O_3_ nanocoating on these flakes not only stabilizes the interface between the active CuO and the electrolyte but also encourages the formation of a stable SEI layer, which is crucial for improving the cycle life of the electrode. When evaluated as anode materials for SIBs, both types of flakes demonstrate impressive electrochemical performance, including high specific capacity and cycling stability. Remarkably, the CuO@Al_2_O_3_ flakes exhibit enhanced high-rate capabilities after the Al_2_O_3_ coating. Furthermore, a full cell fabricated by coupling a CuO@Al_2_O_3_ anode with a NaNi_1/3_Fe_1/3_Mn_1/3_O_2_ cathode also presents high specific capacity and favorable cycling stability. This straightforward and adaptable fabrication approach may hold great promise for the development of a wide range of nano- and micro-structured electrode materials, potentially advancing their applications in high-performance energy storage systems.

## Figures and Tables

**Table 1:** BET surface area and pore size distribution of the CuO and CuO@Al_2_O_3_ flakes.

Samples	BET Surface Area (m^2^ g^−1^)	Average Pore Diameter (nm)
CuO flakes	15.8	16.6
CuO@Al_2_O_3_ flakes	16.1	16.7

**Table 2:** Comparison of sodium storage performance of the as-prepared CuO@Al_2_O_3_ flakes with other CuO-based anodes as reported in the recent literature.

Materials	Rate Capability	Specific Capacity	Voltage Range	References
CuO@Al_2_O_3_ flakes	220 mAh g^−1^ (2000 mA g^−1^)	319 mAh g^−1^ (200 cycles, 200 mA g^−1^)	0.01–3.0 V	This work
CuO micro-nano cubes	218 mAh g^−1^ (800 mA g^−1^)	213 mAh g^−1^ (100 cycles, 100 mA g^−1^)	0.01–3.0 V	[31] *Langmuir* 2024, *40*, 348–361.
CuO/Cu/C nanofibers	120 mAh g^−1^ (2000 mA g^−1^)	300 mAh g^−1^ (400 cycles, 100 mA g^−1^)	0.01–3.0 V	[32] *Rare Metals* 2023, *42*, 4039–4047.
CuO nanocubes	216 mAh g^−1^ (500 mA g^−1^)	170 mAh g^−1^ (100 cycles, 100 mA g^−1^)	0.01–3.0 V	[52] *CrystEngComm* 2021, *23*, 6107–6116.
CuO microspheres	224 mAh g^−1^ (1000 mA g^−1^)	284 mAh g^−1^ (50 cycles, 100 mA g^−1^)	0.01–3.0 V	[53] *Mater. Lett.* 2020, *263*, 127231.
CuO nanoellipsoids	110 mAh g^−1^ (2000 mA g^−1^)	188 mAh g^−1^ (100 cycles, 100 mA g^−1^)	0.01–3.0 V	[27] *ACS Sustainable Chem. Eng.* 2018, *6*, 10876–10885.
CuO nanosheets	230 mAh g^−1^ (2000 mA g^−1^)	394 mAh g^−1^ (20 cycles, 100 mA g^−1^)	0.01–3.0 V	[16] *Mater. Technol.* 2017, *32*, 598–605.
CuO nanoparticles	196 mAh g^−1^ (1500 mA g^−1^)	162 mAh g^−1^ (100 cycles, 100 mA g^−1^)	0.01–3.0 V	[15] *J. Mater. Chem. A* 2016, *4*, 14222–14233.
CuO@C nanofibers	384 mAh g^−1^ (1000 mA g^−1^)	477 mAh g^−1^ (200 cycles, 100 mA g^−1^)	0.01–3.0 V	[55] *Small* 2016, *12*, 4865–4872.

## Data Availability

Data presented in this study are available on request from the corresponding author.
